# Choroidal metastasis from granulocyte colony‐stimulating factor‐producing esophageal squamous cell carcinoma: a case report

**DOI:** 10.1002/ccr3.853

**Published:** 2017-02-23

**Authors:** Shuichi Fukuda, Yoshinori Fujiwara, Hiroshi Mishima, Tomoko Wakasa, Hitoshi Hanamoto, Keisuke Inoue, Kotaro Kitani, Hajime Ishikawa, Masanori Tsujie, Masao Yukawa, Kaoru Okajima, Yoshio Ohta, Masatoshi Inoue

**Affiliations:** ^1^ Department of Gastroenterological SurgeryKindai University Nara HospitalNaraJapan; ^2^ Department of OphthalmologyKindai University Nara HospitalNaraJapan; ^3^ Department of PathologyKindai University Nara HospitalNaraJapan; ^4^ Department of HematologyKindai University Nara HospitalNaraJapan; ^5^ Department of RadiologyKindai University Nara HospitalNaraJapan

**Keywords:** Case report, chemoradiation therapy, choroidal metastasis, esophageal cancer, esophageal squamous cell carcinoma, granulocyte colony‐stimulating factor, leukocytosis

## Abstract

Granulocyte colony‐stimulating factor (G‐CSF)‐producing esophageal squamous cell carcinoma (ESCC) is rare. Esophageal cancer is a highly aggressive disease and often spreads hematogenously; however, choroidal metastases are rarely seen. This report detailed an extremely rare case of G‐CSF‐producing ESCC with choroidal metastasis.

## Background

Granulocyte colony‐stimulating factor (G‐CSF) is a cytokine that contributes to neutrophil production. G‐CSF‐producing tumors have been reported in a variety of organs, such as the lungs, gallbladder, liver, and pancreas 1, 2, 3, 4. However, G‐CSF‐producing esophageal squamous cell carcinomas (ESCCs) have rarely been reported. Esophageal cancer is a highly aggressive disease and rapidly develops metastasis of the lymph nodes 5. Esophageal cancer also often spreads to other organs, such as the lungs, bone, liver, skin, and brain 6; however, choroidal metastases are rarely seen. Here, we report an extremely rare case of G‐CSF‐producing ESCC with choroidal metastasis.

## Case Presentation

A 50‐year‐old Japanese man was referred to our hospital with a chief complaint of dysphagia. The patient reported a 10‐kg weight loss in a few months. The patient also complained of blurred vision, oppressed feeling, and hyperemia in the left eye. The patient had no history of smoking and drinking alcohol. Moreover, the patient had no personal or family history of illness. Physical examination revealed no swelling of superficial lymph nodes. Laboratory data showed an increased white blood cell (WBC) count, 27,100/μL, with 85.0% neutrophils, and increased C‐reactive protein concentration, 12.3 mg/dL. All tumor markers, including carcinoembryonic antigen, fragment of cytokeratin subunit 19, and squamous cell carcinoma‐associated antigen, were within the normal ranges. The serum G‐CSF level was elevated by 60.2 pg/mL (normal level < 39.0 pg/mL). Bone marrow aspiration revealed no possibility of haematological neoplasms.

**Figure 1:**
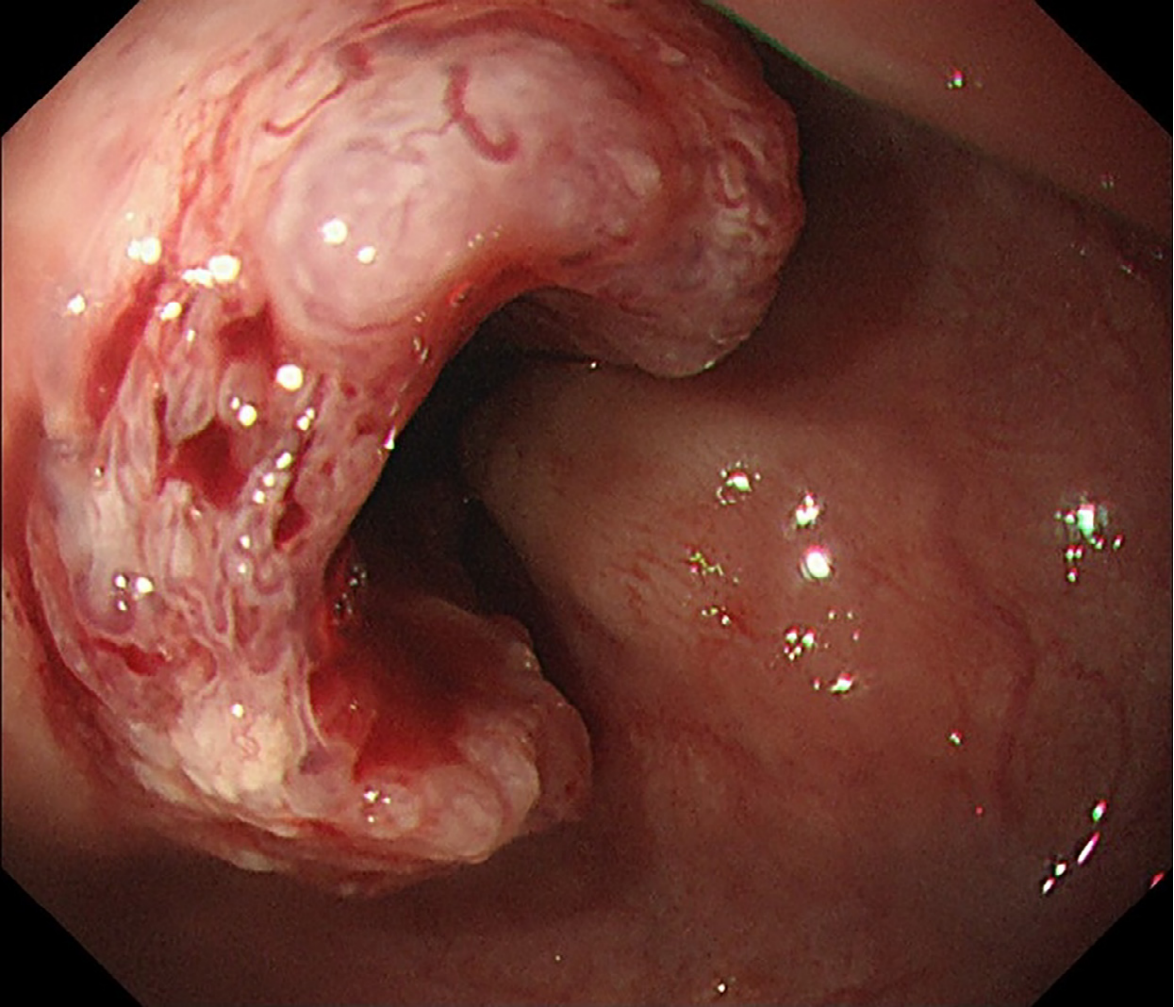
An upper gastrointestinal endoscopy showing a protruding esophageal tumor 30 cm from the incisors extended to the esophagogastric junction.

**Figure 2:**
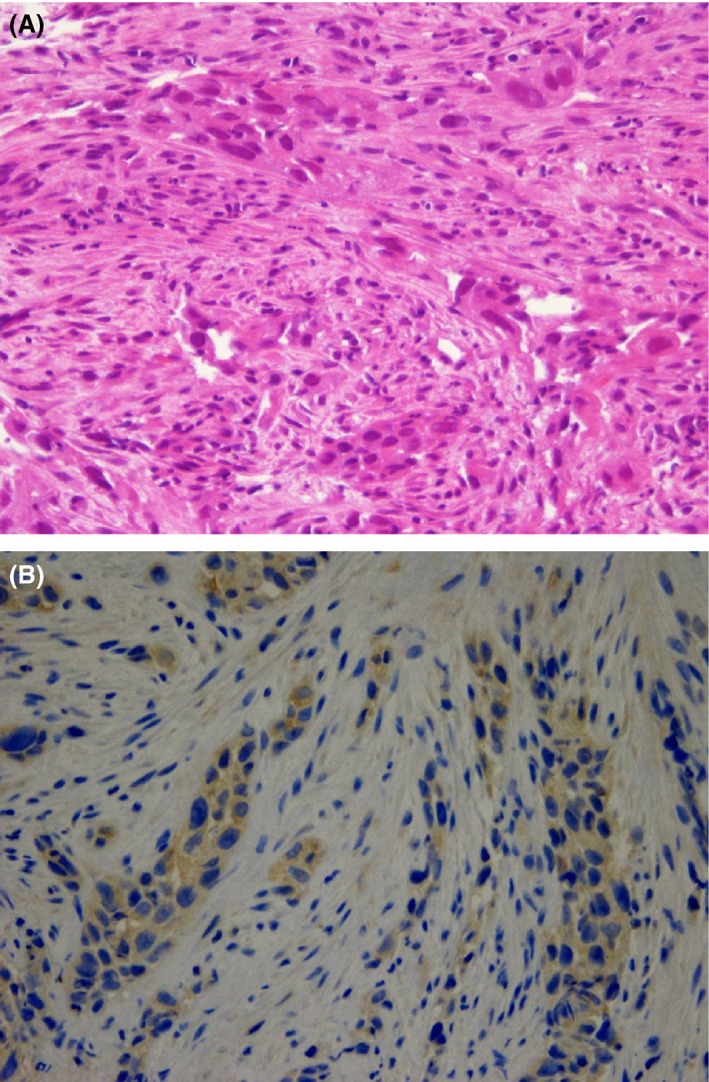
(A) The specimens taken by endoscopic biopsy and histologically confirmed to be poorly differentiated squamous cell carcinoma. (B) Immunohistochemistry showing positive staining for anti‐granulocyte colony‐stimulating factor (G‐CSF) antibody in the cytoplasm of cancer cells.

Contrast‐enhanced computed tomography (CT) from the neck to pelvis revealed thickened wall of the esophagus and several enlarged mediastinal/abdominal lymph nodes. An upper gastrointestinal endoscopy showed a protruding esophageal tumor 30 cm from the incisors extended to the gastric cardia (Fig. 1). The specimens taken by endoscopic biopsy were histologically confirmed to be poorly differentiated squamous cell carcinoma (Fig. 2A). Immunohistochemistry showed positive staining for anti‐G‐CSF antibody in the cytoplasm of cancer cells (Fig. 2B).

**Figure 3:**
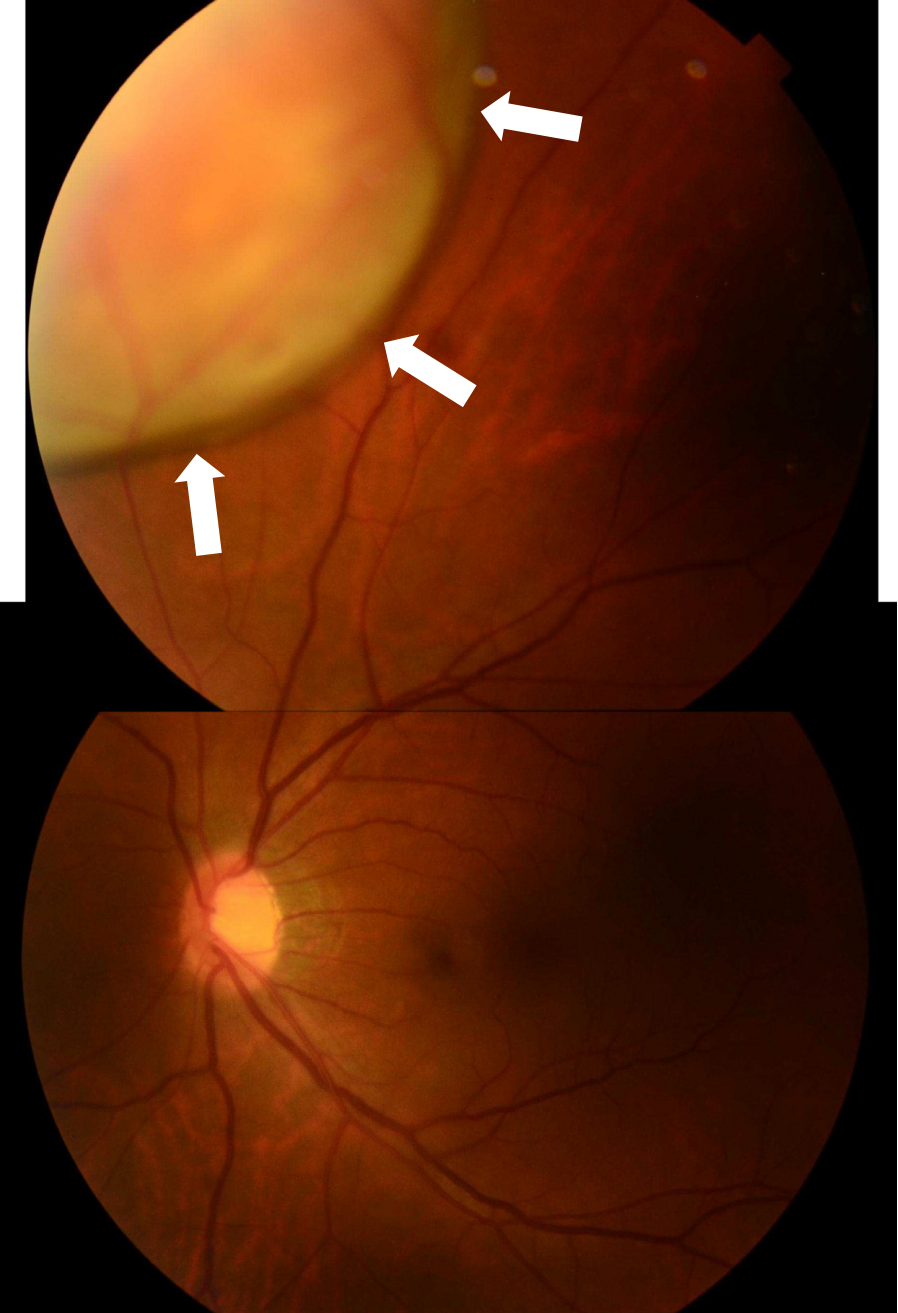
Fundus examination of the left eye showing a well‐circumscribed yellowish‐white choroidal mass at the inner upper side of the posterior pole (arrows).

**Figure 4:**
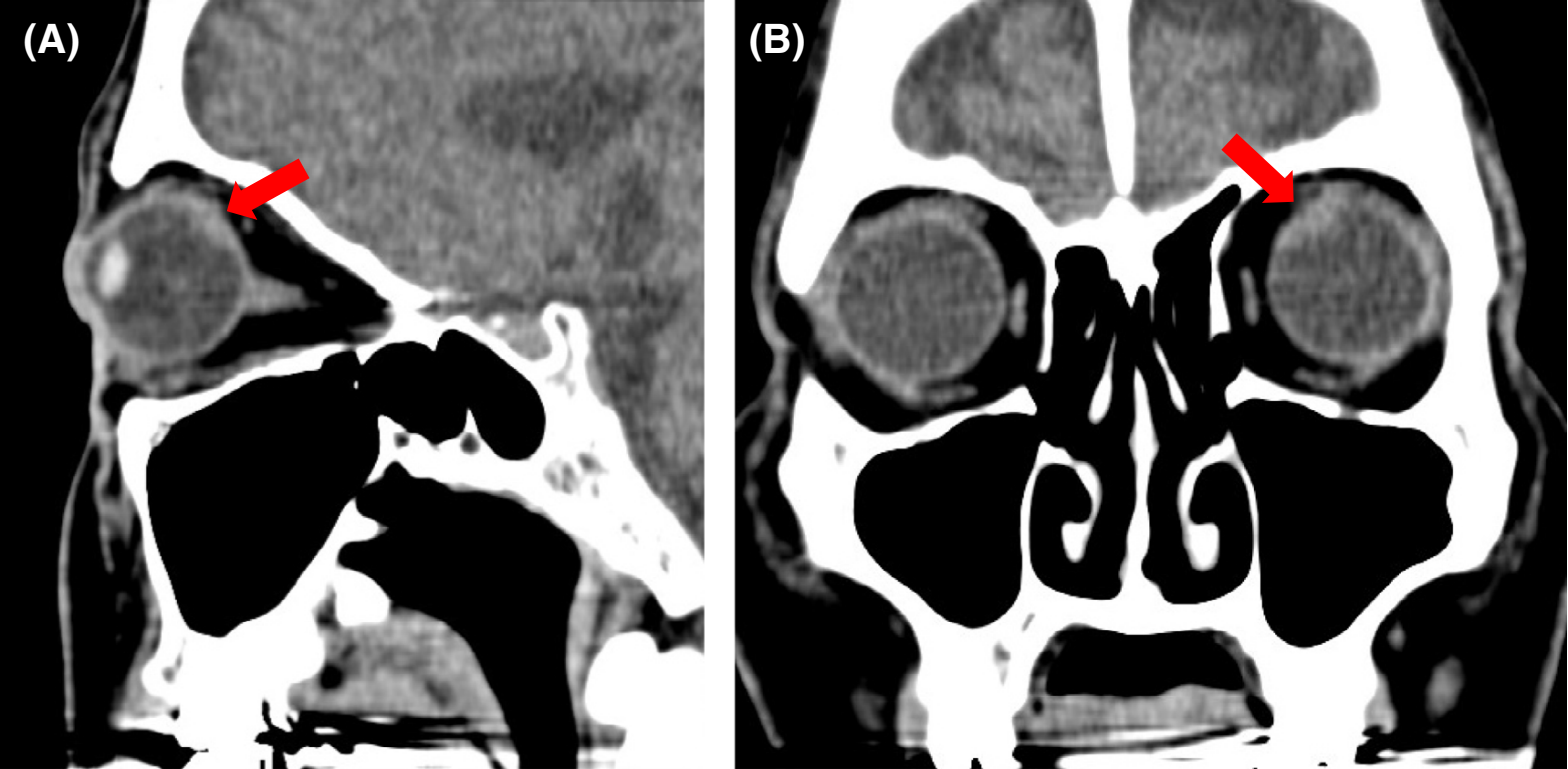
(A, B) Computed tomography showing thickness at the inner upper side of the posterior pole of the left eye.

Ophthalmologic examination revealed that corrected visual acuity and intraocular pressure were normal in the left eye. Fundus examination of the left eye revealed a well‐circumscribed yellowish‐white choroidal mass at the inner upper side of the posterior pole (Fig. 3). CT of the orbit showed thickness at the inner upper side of the posterior pole of the left eye (Fig. 4). Based on these findings, a diagnosis of left choroidal metastasis was made.

The patient was diagnosed with G‐CSF‐producing ESCC T3N2M1, stage IV (according to the Union for International Cancer Control TNM classification of malignant tumors, 7th edition); therefore, radical resection was not recommended. Chemotherapy consisted of cisplatin at 70 mg/m^2^ administered by rapid intravenous infusion on day 1 and 5‐fluorouracil at 700 mg/m^2^ administered by continuous intravenous infusion on days 1 through 5, which was performed with 60 Gy concurrent irradiation in 30 fractions of 2 Gy. Two courses of chemotherapy were performed, separated by a 4‐week interval. A total dose of 30 Gy was also given in 10 fractions of 3 Gy to the left retina, including the right retina for the prevention of metastasis. The treatment was well tolerated, with no grade 3 adverse events.

**Figure 5:**
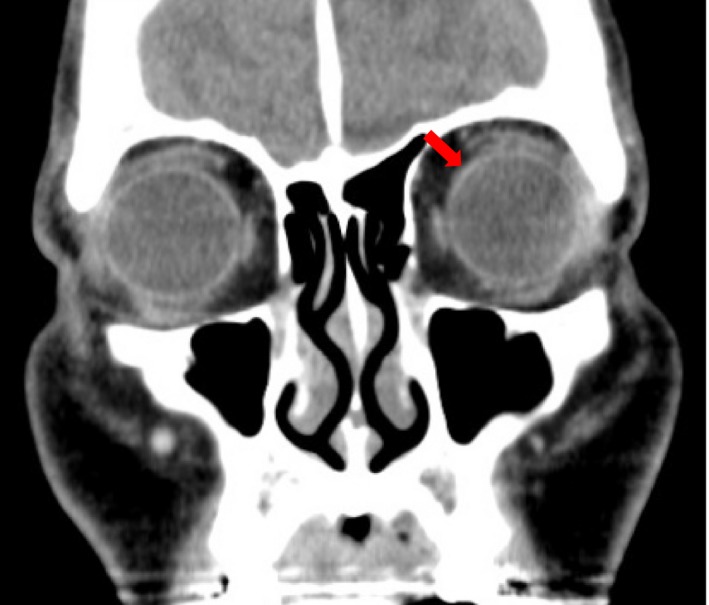
Computed tomography showing that thickness at the inner upper side of the posterior pole of the left eye becoming ambiguous after chemoradiation therapy.

After chemoradiation therapy, the primary tumor of the esophagus decreased, and food intake increased. Moreover, CT showed that thickness at the posterior pole of the left eye became ambiguous (Fig. 5). However, multiple liver metastases soon appeared after chemoradiation therapy, and the patient died 3 months after diagnosis.

**Figure 6:**
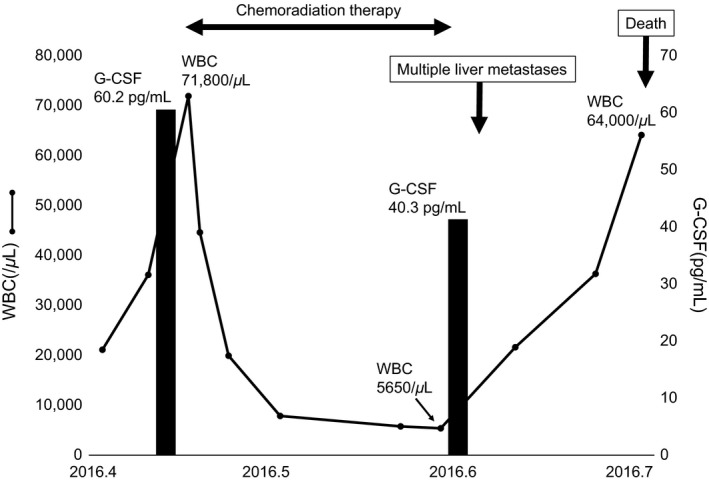
The shift of white blood cell (WBC) count and serum granulocyte colony‐stimulating factor (G‐CSF) level during the treatment.

During the treatment, the WBC count and serum G‐CSF level shifted as shown in Fig. 6. The WBC count reached to 71,800/μL at the start of the chemoradiation therapy and decreased to 5650/μL at the end of therapy. After occurrence of multiple liver metastases, the WBC count increased to 64,000/μL before his death. The serum G‐CSF level decreased from 60.2 to 40.3 pg/mL after the chemoradiation therapy.

## Discussion

Granulocyte colony‐stimulating factor is a cytokine that contributes to production of neutrophils and inducing leukocytosis. G‐CSF is mainly produced endogenously by monocytes, macrophages, mesothelial cells, fibroblasts, endothelial cells, and bone marrow stromal cells 7. Recently, a variety of tumors have been reported to produce G‐CSF 1, 2, 3, 4. G‐CSF‐producing tumors are diagnosed on the basis of elevated serum G‐CSF levels and evidence of G‐CSF production in the tumor 2. In our case, the serum G‐CSF level was elevated, and an immunohistochemical examination showed the cytoplasm of cancer cells to be stained for G‐CSF. Therefore, we diagnosed the patient with G‐CSF‐producing ESCC. To the best of our knowledge, G‐CSF‐producing ESCCs are rare, with only 12 cases reported in English literature, including this report 8, 9, 10, 11, 12, 13, 14, 15, 16, 17, 18. In particular, this report is the first case of G‐CSF‐producing ESCC with choroidal metastasis.

Intraocular metastases of malignant tumors are rare. The choroid is the most common intraocular site for the development of metastases due to the vascular architecture of the choroid and local microenvironmental factors 19, 20. Choroidal metastases have been described frequently for lung and breast cancer 20, 21; in contrast, only a few cases have been reported regarding esophageal cancer 22, 23, 24, 25. Choroidal metastasis is usually diagnosed by ophthalmoscopy and various imaging modalities, such as CT, ultrasonography, and magnetic resonance imaging. However, choroidal metastasis is rarely diagnosed by biopsy because of a risk of seeding tumor cells 19. The common ophthalmologic symptoms are blurred vision, flashes, floaters, and pain; however, some patients have no symptoms 26, 27. The treatment options include chemotherapy, hormonal therapy, radiation therapy, immunotherapy, photodynamic therapy, thermotherapy, and enucleation 19, 26. Choroidal metastasis has an unfavorable prognosis, with a median survival of 6–9 months 19; therefore, the treatment strategy must be chosen carefully, considering the general outcome. In this study, a combination of chemotherapy and radiation therapy was employed, and the choroidal mass decreased without major adverse events.

Previous reports showed that radical tumor resection induced a rapid decrease in both WBC count and serum G‐CSF levels 17, 18. In this study, chemoradiation therapy was performed instead of radical tumor resection, which induced normalization of WBC count. However, the serum G‐CSF level was slightly over the normal level, which would be due to multiple liver metastases. This study suggested that serum G‐CSF level may be a more sensitive metric to discern the viability of cancer cells compared with WBC count.

Table 1 shows 12 cases previously reported in English, including this report. Eleven of the 12 patients (91.7%) were male, with a median age of 66 years (range 30–92 years). None of cases involved the cervical or upper thoracic esophagus. Previous reports seemed to indicate a poor prognosis of G‐CSF‐producing tumors 3, 13; however, it remains unclear whether the prognosis of G‐CSF‐producing ESCC is indeed worse than that of normal ESCC. In fact, three patient cases (cases 4, 5, and 11) in Table 1 without other organ metastasis and multiple lymph node metastasis, in which radical resection was performed, survived more than 18 months. This result suggested that early detection and radical resection may improve survival, even among G‐CSF‐producing ESCCs. G‐CSF‐producing ESCCs are relatively rare as aforementioned; therefore, the number of patients treated in a single institution limits the amount of insight that can be gleaned about the landscape of this rare tumor. The accumulation of prospective evidence from multiple case reports and institutions is needed to clarify the clinicopathological features and adequate treatment strategy of G‐CSF‐producing ESCC.

**Table 1:** Characteristics of G‐CSF‐producing esophageal squamous cell carcinoma

Case	Author	Age	Gender	Location	Differentiation	Stage (TNM 7th)	Therapy	Outcome
1	Ichiishi	66	M	LtAe	Mod to poor	Unknown	Best supportive care	2 months, dead
2	Matsumoto	66	M	Lt	Mod	IIIA–IIIC	Nonradical resection CRT	16 months, dead
3	Kato	54	M	Ae	Mod	IV	Chemotherapy	3 months, dead
4	Komatsu	73	M	LtAeG	Mod	IIB	Radical resection	19 months, alive
5	Nakata	56	M	Lt	Mod	IIB	Radical resection CRT	19 months, alive
6	Mimatsu	69	M	Mt	Poor	IV	Radiation therapy	7 months, dead
7	Tanabe	76	M	LtAe	Mod	IIIC	Radical resection CRT	10 months, dead
8	Mayanagi	30	M	Mt	Well	IIIC	Neoadjuvant CRT Resection	3 months, recurrence
9	Shimakawa	70	M	Lt	Mod	IIIB	Neoadjuvant chemotherapy Radical resection Chemotherapy	12 months, dead
10	Oshikiri	65	M	Lt	Well	IIA	Radical resection	3 months, alive
11	Kitani	92	F	MtLt	Mod	IIIA	Radical resection	18 months, alive
12	Our case	50	M	MtLtAeG	Poor	IV	CRT	3 months, dead

G‐CSF, granulocyte colony‐stimulating factor; Mt, middle thoracic esophagus; Lt, lower thoracic esophagus; Ae, abdominal esophagus; G, stomach; Mod, moderately; Poor, poorly; CRT, chemoradiation therapy.

This report has highlighted an extremely rare case of G‐CSF‐producing ESCC with choroidal metastasis. In the case of an ESCC patient with leukocytosis, the possibility of G‐CSF‐producing cancer should be considered. Moreover, when ESCC patients display ophthalmologic symptoms, we should be aware of potential choroidal metastasis.

## Authorship

SF: designed the study and drafted the manuscript. HM: performed the ophthalmic examination. TW and YO: performed the histopathological examination. HH: performed the haematological examination. KO: made the radiation treatment plan. YF, KI, KK, HI, MT, MY, and MI: participated in the manuscript revision process. All authors read and approved the final manuscript.

## Conflict of interest

None declared.

## Consent for Publication

Written informed consent was obtained from the patient to allow for the publication of this case report and any accompanying images.
